# Effect of Different Drying Methods on Nutrient Quality of the Yellow Mealworm (*Tenebrio molitor* L.)

**DOI:** 10.3390/insects10040084

**Published:** 2019-03-27

**Authors:** Nina Kröncke, Sandra Grebenteuch, Claudia Keil, Sebastian Demtröder, Lothar Kroh, Andreas F. Thünemann, Rainer Benning, Hajo Haase

**Affiliations:** 1 Institute of Food Technology and Bioprocess Engineering, University of Applied Sciences Bremerhaven, An der Karlstadt 8, 27568 Bremerhaven, Germany; nkroencke@hs-bremerhaven.de (N.K.); rbenning@hs-bremerhaven.de (R.B.); 2 Department Food Chemistry and Analytics, Institute of Food Technology and Food Chemistry, TU Berlin, Gustav-Meyer-Allee 25, 13355 Berlin, Germany; sandra.grebenteuch@tu-berlin.de (S.G.); sdemtroeder@hs-bremerhaven.de (S.D.); 3 Department Food Chemistry and Toxicology, Institute of Food Technology and Food Chemistry, TU Berlin, Gustav-Meyer-Allee 25, 13355 Berlin, Germany; c.keil@tu-berlin.de (C.K.); lothar.kroh@tu-berlin.de (L.K.); 4 Federal Institute for Materials Research and Testing (BAM), Unter den Eichen 87, 12205 Berlin, Germany; andreas.thuenemann@bam.de

**Keywords:** *Tenebrio molitor* L., freeze drying, vacuum drying, rack oven drying, fatty acids, volatile compounds, zinc content, bioaccessibility

## Abstract

Yellow mealworm (*Tenebrio molitor* L.) represents a sustainable source of proteins and fatty acids for feed and food. Industrial production of mealworms necessitates optimized processing techniques, where drying as the first postharvest procedure is of utmost importance for the quality of the final product. This study examines the nutritional quality of mealworm larvae processed by rack oven drying, vacuum drying or freeze drying, respectively. Proximate composition and fatty acid profile were comparable between the dried larvae. In contrast, larvae color impressions and volatile compound profiles were very much dependent on processing procedure. High-temperature rack oven drying caused pronounced darkening with rather low content of volatiles, pointing toward the progress of Maillard reaction. On the other hand, vacuum drying or freeze drying led to enrichment of volatile Maillard reaction and lipid oxidation intermediates, whose actual sensory relevance needs to be clarified in the future. Beyond sensory and visual importance drying intermediates have to be considered with regard to their metal ion chelating ability; in particular for essential trace elements such as Zn^2+^. This study found comparable total zinc contents for the differently dried mealworm samples. However, dried larvae, in particular after rack oven drying, had only low zinc accessibility, which was between 20% and 40%. Therefore, bioaccessibility rather than total zinc has to be considered when their contribution to meeting the nutritional requirements for zinc in humans and animals is evaluated.

## 1. Introduction

Recent predictions of the United Nations assume that the global human population will exceed the 11 billion threshold by the year 2100 [1]. These developments challenge us to broaden the view for alternative nutritional resources. Insects are increasingly becoming the focus of attention due to their high content of proteins, unsaturated fatty acids and minerals [2,3]. The yellow mealworm (*Tenebrio molitor* L., Coleoptera: Tenebrionidae) is an edible insect and due to its ubiquitous occurrence and the frequency of consumption, a promising candidate for the cultivation and production on an industrial scale [2,4]. Mealworm larvae represent a very sustainable source for feed and food because of their high nutrient value with rather low contaminant levels [5,6]. Mass rearing of livestock—such as beef, pork or chicken—has several negative environmental impacts; including high energy, land, water demand, and extensive generation of ammonia and greenhouse gases [7]. The latter problem could be substantially diminished in case of mealworm replacement [8]. From an energetic point of view, requirements for protein production are almost comparable between mealworms or beef/pork; but relative land requirements were estimated to be almost 3–15 fold lower in case of mealworms [9]. Water footprint per edible ton of mealworms is comparable to chicken meat (4341 m^3^/t) but 3.5 times lower than the water footprint of beef [10]. Still, when provided with an optimal diet, the feed conversion efficiency of mealworms is much better than for livestock [11], making *Tenebrio molitor* L. a promising candidate for food and feed production.

However, the high water content (up to 68%) and water activity increase fresh larvae susceptibility for enzymatic and non-enzymatic degradation and microbiological spoilage. Likewise, Maillard reaction, a chemical reaction between amino components and reducing sugars causing coloring and formation of flavor- and redoxactive compounds, is facilitated in the presence of water as well [12,13,14,15,16,17]. Thus manufacturers are challenged to find appropriate drying technologies in order to preserve the insects for extended periods of storage without any loss in nutrient and sensory quality. Mealworms are generally rich in fats (between 10% and 30% in dried matter) and do have high amounts of unsaturated fatty acids, which makes them vulnerable to lipid oxidation and formation of hardly soluble lipid-protein adducts during drying and storage [12,18]. In addition to macronutrients, the accessibility of essential micronutrients from insect matrices might be affected, too. Consumption of edible insects is outstandingly promoted to improve the human micronutrient status, including that of essential trace elements such as iron or zinc [19,20,21,22]. The mineral content for a variety of insects has already been summarized recently [23]. However, knowledge on micronutrient accessibility from this matrix is rather poor, in particular with regard to the insect processing status [21,24,25]. Beside nutritive aspects, drying procedure has a huge impact on sensorial quality of the dried product. Formation of volatile compounds, such as aldehydes, ketones or alcohols in the course of drying designates flavoring properties of the product, either desirable or undesirable for consumers. However, the variety of precursors present in food matrices and thus the plurality of reaction options hardly allows predicting a product’s sensory properties when being processed, making empirical investigations indispensable. In western communities, including Europe and the US, adjustable drying methods, like evaporation, drum drying, freeze- and spray-drying, are common in industrial practice to process and preserve a variety of animal- and vegetable-based food products [24,26]. 

Drying of insects is of the utmost importance within the entire insect processing chain and thus needs to be done properly to reduce potential microbial, chemical and allergenic hazards, while maintaining nutritive properties and acceptance by consumers [3,4,19]. For *Tenebrio molitor,* recent publications already stated the effect of certain drying procedures on coloring, macronutrient composition, protein solubility and lipid oxidation prevalence of mealworm larvae [12,27,28]. Within the current study we aimed to compare fatty acid and volatile component profiles of late instar larvae upon processing by either freeze drying, vacuum oven or rack oven drying. Along with effects on total zinc content and bioaccessibility of this essential trace element, these results will contribute to improving *Tenebrio molitor* processing technologies, a basic prerequisite for utilizing mealworms as novel food or animal feed in the future.

## 2. Materials and Methods

### 2.1. Insect Samples

*Tenebrio molitor* larvae used in this study were cultured at the University of Applied Sciences Bremerhaven (Bremerhaven, Germany). The larvae were kept in a rearing room at 25 °C with a relative humidity of 55–60% and were fed ad libitum with wheat bran. Mealworms were reared for a period of 16–18 weeks until harvest. At harvest time, larvae were separated from wheat bran and frass and frozen at −21 °C for 48 h (HAS 47520, Beko, Neu-Isenburg, Germany) in a stainless steel container (53 × 32.5 × 4 cm) before drying. The larvae rearing and the experiments were carried out under full observance of the German Animal Welfare Act.

### 2.2. Experimental Setup

In the present study, *Tenebrio molitor* larvae were processed by either freeze drying, vacuum oven drying or rack oven drying as described previously [27]. For freeze drying frozen samples of 100 g of larvae were subdivided with a stainless steel dosing scoop (Contacto, Erkrath, Germany) onto five freezing plates (diameter: 36 cm) and placed within a freeze dryer (Christ Beta 1–8, Martin Christ, Osterode am Harz, Germany). Before starting the drying process, the condenser was set to −50 °C and a vacuum was applied for 24 h with final temperatures of around 20 °C at the shelves. The vacuum oven (VT 5042, Heraeus, Hanau, Germany) was set to 60 °C and 300 g of larvae were dried on two metal sheets (41 × 30 × 2 cm) for 24 h under vacuum. A rotating rack oven (RI 1.0608-TL, MIWE, Arnstein, Germany) was used to dry a thin layer of 500 g of larvae on a baking plate (60 × 80 × 2 cm) in the middle of the rotating oven at 120 °C for 1h at ventilation stage 2. After drying, the larvae were separately packed in closed polyethylene bags and stored for a week at 5 °C in a climatic chamber (HPP 110, Memmert, Schwabach, Germany).

### 2.3. Proximate Analysis

*Tenebrio molitor* larvae were analyzed for proximate composition as described previously [27]. The moisture content was determined after drying homogenized larvae in a drying oven (U10, Memmert, Schwabach, Germany) for 4 h at 103 °C. Protein content was determined by the Kjeldahl method and was calculated by multiplying the measured nitrogen content with a factor of 6.25 according to DIN EN 25663 and the Association of German Agricultural Analytic and Research Institutes [29]. Total fat content was determined according to the Soxhlet method (VDLUFA, 1976). Fibre and ash content were analyzed as described by VDLUFA, 1976 [29].

### 2.4. Color Evaluation

Color evaluation was done from digital images according to a protocol from de Oliveira et al. [30]. Digital pictures from dried larvae samples were evaluated with Photoshop CS4 Version 11.0 (Adobe, San José, CA, USA). Following background correction, the larvae bunch was scaled to 800*800 pixels and 5 different areas per bunch marked by the eyedropper tool to 101*101 pixels analyzed for CIE L*A* B* parameters (a color space defined by the International Commission on Illumination (CIE)). These coordinates are the lightness of the color, where L* = 0 yields black and L* = 100 indicates diffuse white. The values of components a* (green-red axis) and b* (blue-yellow axis) are between −128 and +128 respectively. Based on these values, total color differences (ΔE*) between mealworm samples were calculated with $\Delta E=\sqrt{a^{*2}+b^{*2}+c^{*2}}$ [12].

### 2.5. Fatty Acid Composition

Lipids were extracted from mealworm larvae samples by methanol/chloroform extraction prior to trimethylsulfonium hydroxide derivatization [31]. The fatty acid composition was measured using a gas chromatography–flame ionization detection system (GC-FID) (GC-2025, Shimadzu, Duisburg, Germany) with a DB-23 column (60 m × 0.25 mm × 0.25 µm; Agilent Technologies Deutschland GmbH, Munich, Germany) applying helium as carrier gas (1.15 mL/min). The following temperature program was used: initial rise in temperature from 130 °C to 180 °C (10 °C/min) followed by a rise to 220 °C (5 °C/min) then hold for 17 min. Fatty acid methyl esters were identified by comparison of their retention times with fatty acid methyl ester standards (Sigma Aldrich, Munich, Germany).

### 2.6. Volatile Compounds

Volatile compounds from mealworm larvae samples were analyzed by static headspace gas chromatography-mass spectrometry, already applied for various food matrices before [32]. Then, 1 g of mealworm samples were weighed into 20 mL headspaces and incubated at 100 °C for 15 min to carry volatile compounds into the vapor phase. Subsequently, 1 mL of vapor space was injected into a gas chromatography-mass spectrometry (GC-MS) system consisting of a GC-17A gas chromatograph (Shimadzu, Duisburg, Germany) with a Rtx-Volatiles column (60 m × 0.25 mm, 1 μm) and MS-QP5000 mass detector (Restek, Bad Homburg, Germany). The following temperature program was used: initial temperature 40 °C for 5 min, initial increase in temperature to 150 °C (10 °C/min) up to the final temperature of 210 °C (2 °C/min). Chemical identification was done by comparing retention times and mass spectra of samples with those of commercial standards (Sigma-Aldrich, Munich, Germany) and data available from NIST library (National Institute of Standards and Technology, United States).

### 2.7. Total Zinc Content and Bioaccessibility

Defined amounts of *Tenebrio molitor* larvae were subjected to a microwave-assisted digestion (Mars 6, CEM GmbH, Kamp-Lintfort, Germany) with a 1:1 mixture of ultrapure HNO_3_ (65%) and H_2_O_2_ (30%). Zinc content was analyzed by flame atomic absorption spectrometry (FAAS) on a Perkin Elmer AAnalyst 800 (Perkin Elmer, Rodgau, Germany) applying an external calibration (analytical parameters: LOD 10.3 µg Zn/L; LOQ 15.9 µg Zn/L). For determination of zinc accessibility, mealworm larvae underwent a simulated human in vitro digest as described before [33]. Briefly, 2 g of mealworms were stirred in 7.5 mL synthetic saliva for 5 min before acidification (pH 2.0) and treatment with 17.5 mL of artificial gastric juice for 2 h. Thereafter mixtures were neutralized by powdery sodium bicarbonate and incubated for another 2 h in 25 mL intestinal juice, before separation of insoluble components by centrifugation. The integrity of the amylolytic, proteolytic and lipolytic enzymes was verified during digestion process using distinct control substrates for each of these hydrolases. Digested larvae samples were subsequently also handled by microwave-assisted digestion and FAAS applied for zinc quantification. Relative Zn-bioaccessibility (BA_Zn_) was calculated with BA_Zn_ = (Zn_Supernatant_/Zn_total_) × 100%.

### 2.8. Statistical Analysis

Statistical significance of the experimental results was calculated by GraphPad prism software (version 5.0, GraphPad Software, San Diego, CA, USA). Data were compared between treatments using one-way ANOVA with Bonferroni’s multiple comparison test. A 95% confidence level was presumed.

## 3. Results

### 3.1. Proximate Composition

Mealworms grown until late instar larvae stage were dried by either freeze drying, vacuum oven drying or rack oven drying and assessed for proximate composition (see Table 1). Depending on the drying strategy, different residual moisture contents were measured, with the highest levels of around 9.83% for freeze dried samples. Any of the other parameters—such as protein, fat and fiber content-were very similar, despite small fluctuation ranges. Thus, dried mealworm larvae from a proximate composition perspective were comparable in quality to other crude or processed insects [12,19,23,24,34].

### 3.2. Color Analysis

Color measurements for the dried mealworm larvae are shown in Table 2. In direct comparison with the data given by Lenaerts et al. [12] our freeze dried samples were higher in L* value (lighter) and slightly more positive in red (a*) and yellow (b*) components, which might be due to shorter duration of freeze drying. When ranking this study’s drying procedures, rack oven drying led to mealworm larvae with the strongest darkness. High temperatures of around 120 °C inside the rack oven favors non-enzymatic browning, leading to formation of colored Maillard products most likely responsible for lower lightness. Based on the estimated total color differences, freeze-dried mealworm larvae were more similar in color to rack oven dried samples (ΔE*-value 11.2) than freeze dried *Tenebrio molitor* L. (ΔE*-value 17.9).

### 3.3. Fatty Acid Analysis

For fatty acid analysis, dried larvae were subjected to Folch-based chloroform/methanol extraction, already proven in efficiency for various food matrices including insects [35,36], esterification and GC−FID analysis.

The composition of each of the dried samples (see Table 3 and Figure S1A,B) is very similar to the pattern already reported for either crude or dried *Tenebrio molitor* L. [12,23,35,37,38]. Fatty acids of C_14_ to C_18_ chain length were detected, with no evidence for C_20_ or even higher elongated molecules (see Table 3 and Figure S1B). Overall linoleic acid, oleic acid and palmitic acid are the major fatty acid components in quantity. Albeit the samples slightly differed in content for some of the fatty acids, the high level of linoleic acid (~35% of total fatty acids) led to a polyunsaturated to saturated fatty acid (P/S) ratio close to 1.6 each, favourable with regard to dietary recommendations [39,40].

### 3.4. Volatile Components

**Figure 1:**
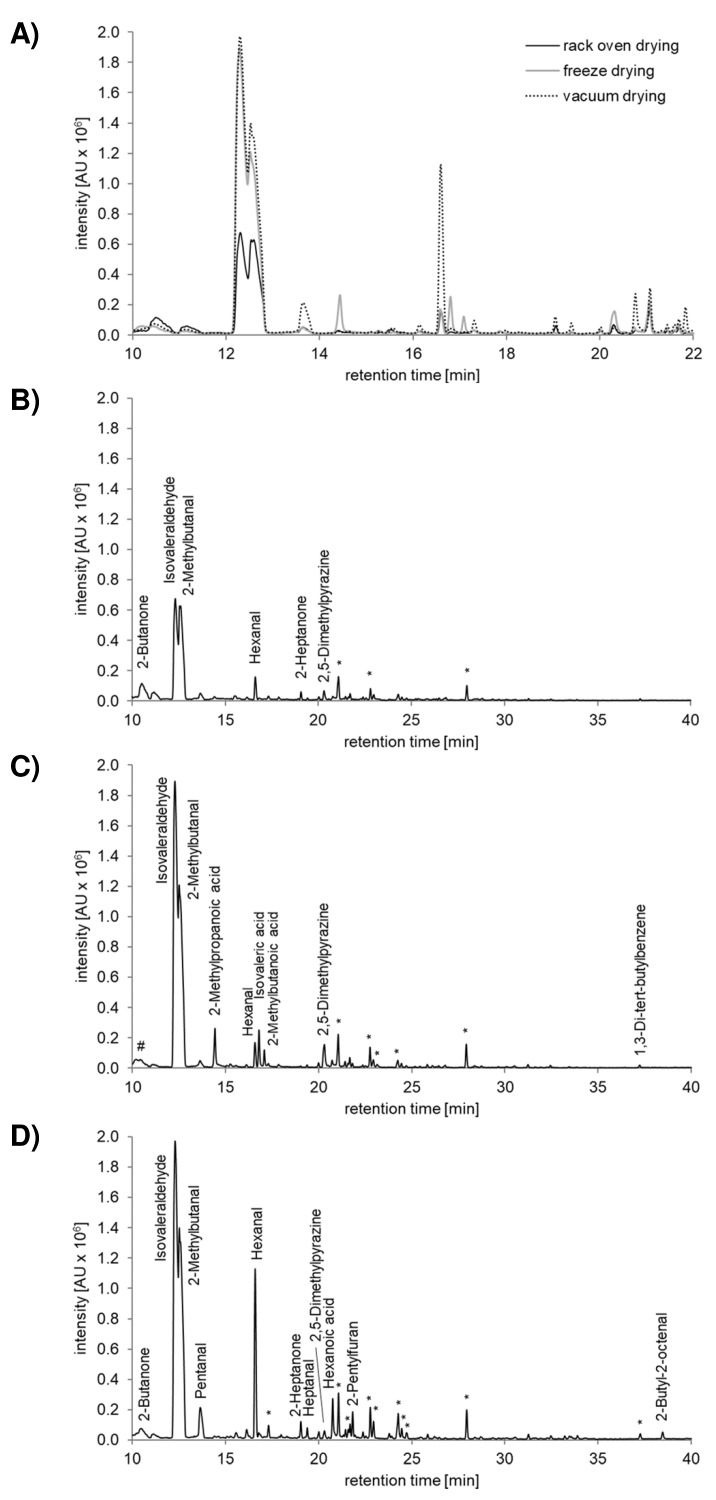
Headspace gas chromatographicanalysis of dried *Tenebrio molitor* larvae. Representative GC-chromatograms out of three independent head space GC−MS experiments are shown in overview (**A**) and in detail for rack oven drying (**B**), freeze drying (**C**) and vacuum drying (**D**). * alkanes < 0.002% w/w, # mixed peak of 2-butanon and diacetyl.

A direct comparison of the GC-MS profiles outlined differences between the three drying procedures, with vacuum-dried or freeze dried samples being much more diverse in molecule composition (see Figure 1A). Intermediates of both Maillard reaction (isovaleraldehyde; isovaleric acid; 2-methylbutanal; 2,5-dimethylpyrazine; 2-methylbutanoic acid; 2-methylpropanoic acid) and lipid oxidation (2-butanone; 2-butyl-2-octenal; 2-heptanone; 2-pentylfuran; heptanal; hexanal; hexanoic acid; octanal; pentanal; 2-pentylfuran) were detected (Figure 1B–D, Table S1). Noteworthy is the comparatively high content of alkanes, also detectable in wheat bran (Figure S2B), most likely accumulating within the larvae in the course of drying. The two strecker aldehydes isovaleraldehyde and 2-methylbutanal were already present in crude larvae (Figure S2A) but seemed to be higher in quantity in vacuum-dried or freeze-dried larvae, suggesting that they are increasingly formed during drying. However, GC-MS analyses from crude and dried larvae were done from different batches so conclusions have to be made carefully. In tendency the GC-MS profile from vacuum-dried larvae was more enriched in aldehydes and acids formed in the course of lipid oxidation. Instead the strecker degradation products, 2-methylbutanoic acid, isovaleric acid, 2-methylpropanoic acid and 2,5-dimethylpyrazine, were typical for freeze dried samples, with relatively low amounts of secondary lipid oxidation products (Figure 1C,D, Table S1).

### 3.5. Zinc Concentration and Bioaccessibility

**Figure 2:**
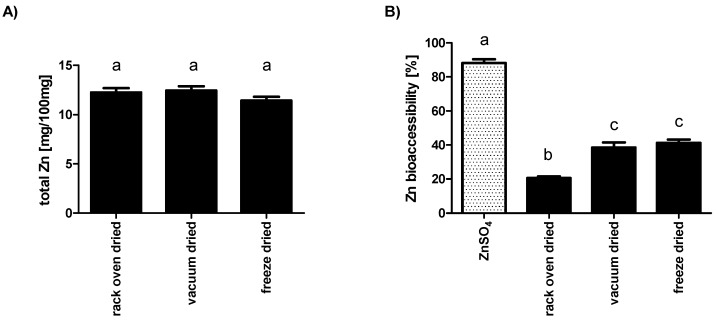
Total amount and bioaccessibility of zinc from dried *Tenebrio molitor* larvae. Mealworm larvae were analyzed for zinc content before or after incubation with gastrointestinal digestive secretions using flame atomic absorption spectrometry. The total zinc content (**A**) as well as Zn bioaccessibility (**B**) were calculated. Data are shown as means ± SEM of three replicates. Significantly different means do not share the same letters (analyzed by one-way ANOVA with Bonferroni’s multiple comparison test).

Figure 2A shows the absolute zinc content for *Tenebrio molitor* larvae. Overall the levels were almost comparable between the differently dried mealworm samples. The estimated values of around 12 mg/100 g weight is very similar to the concentrations previously reported for crude mealworm larvae [21,23,41], suggesting no significant loss of the trace element in the course of drying.

From a nutritive perspective the bioaccessibility, i.e., the amount of zinc enzymatically released and potentially available for absorption, is far more relevant than the absolute mineral content [42]. Therefore, dried larvae or an equal amount of ZnSO_4_, a control for the availability of inorganic zinc, were treated with digestive enzymes mimicking the human gastrointestinal tract [33], prior to quantification of total soluble zinc species. Almost all of the inorganic zinc was present in the soluble fraction upon in vitro digestion (Figure 2B); minimal losses may be due to binding to mucins [43] included in the intestinal juice. Instead, more than 60% of the total zinc from vacuum-dried and freeze dried or even 80% from rack oven dried samples remained inaccessible (Figure 2B).

## 4. Discussion

Global population growth will increasingly challenge industry over the coming years. Consequently, the interest in insects as an alternative environmentally friendly food and feed source is constantly rising [19] and there is considerable pressure to specify and harmonize rules on safety, marketing and animal farming [4,44,45]. However, especially in western cultures, the attitude toward insects is rather reluctant—mainly for psychological reasons—and willingness to practice entomophagy is low. Prospectively, insect processing is clearly the trend to overcome uncertainty and disgust, which are the greatest problems [46,47]. Out of the 1000 to 2000 edible insect species worldwide [48], *Tenebrio molitor* L. is a promising candidate for food and feed production [4] and almost any farming and processing step is addressed from economical, but also from food safety and nutritional quality perspectives [3,19]. The current study confirmed and underlined our recent observations on the efficiency of freeze drying, vacuum oven drying and rack oven drying with regard to moisture reduction of *Tenebrio molitor* [27], which is crucial to restrict microbial spoilage [13,48]. Depending on the drying method, the residual moisture content ranged from 0.87 to 9.83%, which is sufficient for long term storage [49]. Even though dehydration kinetics was not measured herein, the high moisture content of fresh larvae with a water activity close to 1 [27] favors lipid oxidation and non-enzymatic browning reactions already during the drying process [17]. Lipid peroxidation products were previously detected in dried mealworm samples, where total values differed depending on the drying technique applied [12,27]. By analyzing volatile components, the present study now provides first insights into both branches, Maillard reaction and lipid oxidation, and the results underpin the relevance of the drying procedure. Volatile profile from rack oven dried mealworm larvae was comparatively low in Maillard and fat oxidation intermediates. It is quite likely that high temperatures of around 120 °C in the rack oven expedite non-enzymatic browning, whereby the emergence of terminal, antioxidative Maillard reaction products restrains lipid oxidation [50]. This assumption is corroborated by two observations: first this study’s color evaluation showing the highest darkness for rack oven dried larvae and second the much lower lipid peroxidation status indicated by 4-hydroxy-2-nonenal (4-HNE) quantification [27]. Vacuum dried larvae were slightly higher in 4-HNE content in the aforementioned investigations and this study’s volatile screening matches that observation showing GC-MS profiles enriched in lipid oxidation aldehydes and acids. It is difficult to assess the actual sensory relevance of the detected compounds. Almost all of them were characterized with regard to their sensory properties and the odor threshold values within the past [51,52,53]. However, included in complex food matrices these odorants may lose their individuality and rather contribute, by cooperation with other components, to the unique odor quality of the product [54]. For example, studies analyzing cooked tail meat of American lobster by chromatography—olfactometry techniques (sniffing GC)—observed that isovaleraldehyde separately has a typical chocolate-malty-like odor, but as part of the total matrix contributes to the characteristic overall aroma of cooked lobster tail meat [55]. Similar experiments are urgently needed to assess odor active compounds of dried mealworm larvae, an important step toward advancing the processing of insects in terms of sensory acceptance.

For humans, dietary micronutrients, including the trace element zinc, are essential for proper physiological functioning and they have to be provided by nutrition in sufficient amounts [56,57,58]. Edible insects are generally rich in zinc content, but biochemical studies examining zinc speciation in insect tissues or cells are comparatively rare [21]. Based on the quantification of total zinc levels 100 mg from any of the dried mealworm larvae would be more than sufficient to meet current reference values for daily dietary zinc intake of 7–10 mg [59,60,61]. However, the relatively low bioaccessibility necessitates a much higher supply to replenish the daily zinc losses [60]. When questioning the reasons for decreased accessibility the zinc chelator phytate [62,63] falls well below harmful concentrations within insects [46], thus being out of concern. Far more relevant are chitin molecules, long-chain N-acetylglucosamine polymers present in insects including *Tenebrio molitor* [28,64], already known to bind metal ions, including Zn^2+^, in high quantities [65]. Importantly, chitins are almost indigestible by human gastrointestinal tract (GIT) hydrolases [46,66], which might, at least in part, explain the relatively low zinc accessibility from dried mealworm larvae observed in the present study. However the observation of a distinctly lower zinc release from rack oven dried larvae in comparison to larvae dried by the other two methods indicates that heating-induced molecules are of importance as well. In this respect, the zinc-binding ability of late stage Maillard products needs to be mentioned [67], which has already been shown to be of importance for intestinal zinc resorption [68,69]. A worthwhile strategy for improving zinc accessibility from mealworm larvae could be the use of Zn-enriched feed during breeding. The results of a very recent study indicate elevated zinc levels in Zn-fed mealworm larvae. Hereby, an increase not just in total zinc content, but also within the soluble, cytosolic fractions was observed [70]. In future experiments, this kind of Zn-enriched *Tenebrio molitor* L. should be applied to combined in vitro digestion cell culture model systems to study zinc resorption under human GIT-like conditions [42,71], or even tested in human trials [72] to evaluate its bioavailability. Moreover, as zinc is a well-known pro-antioxidant [73], Zn-biofortification might even improve stability of *Tenebrio molitor* during processing and storage by elevating various antioxidant mechanisms, which would be of utmost important from both an economical and a nutritive perspective.

## 5. Conclusions

Edible insects such as *Tenebrio molitor* larvae are often promoted as an auspicious alternative to conventional protein sources, but knowledge on processing-induced changes in their nutrient quality is still scarce. Hence, studies aiming at the improvement of industrial-scale processing are required, in order to process insects in the best possible way, from both nutritive and economics points of view. Summarizing this study’s results, it can be concluded that late stage instar mealworm larvae upon processing by either freeze drying, vacuum oven or rack oven drying, respectively, were similar in proximate composition and fatty acids profiles. Rack oven drying progressing Maillard reaction is favorable with regard to the oxidative stability of mealworm larvae, but had the strongest inhibitory effect on enzymatic release of the essential trace element zinc under human GIT-like conditions. On the other hand, freeze-dried or vacuum-dried larvae yielded far more diverse product spectra of volatile Maillard reaction and lipid oxidation intermediates, whose sensory relevance needs to be clarified. When assessing the drying process, the costs of drying should also be taken into account. The costs of drying were calculated for all methods. The calculation is based on the assumption that the equipment runs at maximal capacity during drying. Energy cost was highest for vacuum oven dried mealworms at € 3.24/kg, followed by freeze drying with € 2.88/kg. The process time was generally much shorter for rack oven drying; consequently the energy costs of € 0.67/kg are much lower than freeze drying and vacuum oven drying. Considering the energy cost in relation to the quality of dried larvae, long process times should be avoided. Overall these results are a further step forward in the current development of process-optimized drying strategies in order to be able to process *Tenebrio molitor* optimally in the future, both in terms of nutrient quality and under quantitative aspects.

## Figures and Tables

**Table 1:** Nutritional values of fresh and dried mealworm larvae.

Parameter	Before Drying	Rack Oven Dried	Vacuum Dried	Freeze Dried
Moisture (g/100g)	62.87 ± 0.27 ^a^	0.87 ± 0.03 ^b^	1.70 ± 0.09 ^c^	9.83 ± 0.03 ^d^
Protein (g/100 g DM)	53.53 ± 0.28 ^a^	56.30 ± 0.32 ^b^	53.23 ± 0.20 ^a^	52.23 ± 0.19 ^a,c^
Fat (g/100 g DM)	27.13± 0.03 ^a^	27.27 ± 0.09 ^a^	29.57 ± 0.02 ^b^	26.80 ± 0.06 ^c^
Fibre (g/100 g DM)	6.47 ± 0.09 ^a^	7.10 ± 0.06 ^b^	6.83 ± 0.03 ^b,c^	7.53 ± 0.09 ^d^
Ash (g/100 g DM)	3.27 ± 0.12 ^a^	3.43 ± 0.18 ^a^	3.40 ± 0.15 ^a^	3.43 ± 0.12 ^a^

DM = mass of dried mealworms; Data are shown as means ± SEM of three replicates. Significantly different means within one row do not share the same letters (analyzed by one-way ANOVA with Bonferroni’s multiple comparison test).

**Table 2:** Color parameters of rack oven dried, vacuum dried and freeze dried mealworm larvae.

Color Value	Rack Oven Dried	Vacuum Dried	Freeze Dried
L*	36.93 ± 0.10 ^a^	49.73 ± 0.82 ^b^	47.53 ± 1.12 ^b^
a*	15.87 ± 0.26 ^a^	20.08 ± 0.40 ^b^	14.40 ± 0.28 ^c^
b*	29.93 ± 0.58 ^a^	41.40 ± 0.46 ^b^	33.20 ± 0.74 ^c^

Data are shown as means ± SEM of three replicates. Significantly different means within one row do not share the same letters (analyzed by one-way ANOVA with Bonferroni’s multiple comparison test).

**Table 3:** Fatty acid composition of rack oven dried, vacuum dried and freeze dried mealworm larvae.

Fatty Acid	% (Total Fatty Acids)		
	Rack Oven Dried	Vacuum Dried	Freeze Dried
Myristic acid (C14:0)	2.61 ± 0.05 ^a^	2.87 ± 0.05 ^a^	2.20 ± 0.08 ^b^
Palmitic acid (C16:0)	18.08 ± 0.30 ^a^	21.89 ± 0.76 ^b^	17.41 ± 0.39 ^a,c^
Palmitoleic acid (C16:1)	1.95 ± 0.01 ^a^	1.57 ± 0.04 ^b^	1.42 ± 0.02 ^c^
Stearic acid (C18:0)	2.70 ± 0.09 ^a^	4.27 ± 0.26 ^b^	3.52 ± 0.05 ^b^
Oleic acid (C18:1)	36.56 ± 0.35 ^a^	32.93 ± 1.11 ^a^	36.07 ± 0.74 ^a^
Linoleic acid (C18:2)	36.44 ± 0.63 ^a^	34.99 ± 0.25 ^a^	37.66 ± 1.12 ^a^
Linolenic acid (C18:3)	1.64 ± 0.06 ^a^	1.48 ± 0.03 ^a^	1.66 ± 0.10 ^a^
P/S ratio	1.63 ± 0.06	1.80 ± 0.06	1.71 ± 0.09

P/S = Polyunsaturated/saturated fatty acids; data are shown as means ± SEM of three replicates. Significantly different means within one row do not share the same letters (analyzed by one-way ANOVA with Bonferroni’s multiple comparison test).

## Supplementary Materials

The following are available online at https://www.mdpi.com/2075-4450/10/4/84/s1, Figure S1: Fatty acid spectrum of processed, Figure S2: GC-chromatograms form wheat bran (A) and *Tenebrio molitor* Larvae (B), Table S1: Tabular representation of Headspace GC-analysis of dried *Tenebrio molitor* larvae.
