# Supplementary material for: Effect of Different Drying Methods on Nutrient Quality of the Yellow Mealworm (Tenebrio molitor L.)
Source: Insects. 2019 Mar 27;10(4):84. doi: 10.3390/insects10040084 (PMC6523706; doi:10.3390/insects10040084)
Supplement: Supplementary file 1 [file insects-10-00084-s001.pdf]

Supplementary Materials: Effect of Different Drying Methods on Nutrient Quality of the Yellow Mealworm (*Tenebrio molitor* L.)

Nina Kröncke, Sandra Grebenteuch, Claudia Keil, Sebastian Demtröder, Lothar Kroh, Andreas F. Thünemann, Rainer Benning, Hajo Haase

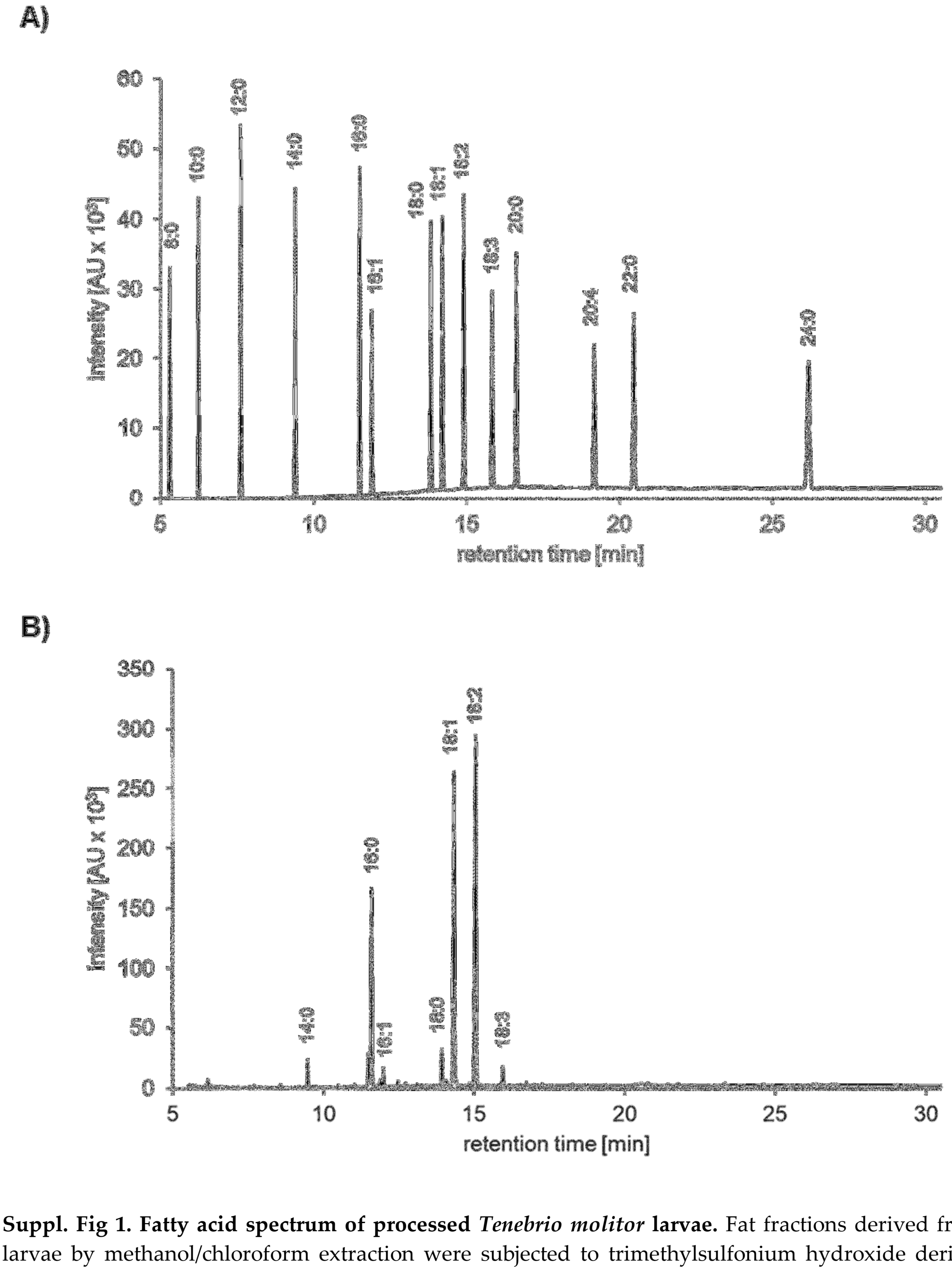

Suppl. Fig 1. Fatty acid spectrum of processed *Tenebrio molitor* larvae. Fat fractions derived from mealworm larvae by methanol/chloroform extraction were subjected to trimethylsulfonium hydroxide derivatization and analyzed for their fatty acid composition by GC-FID. Representative GC-chromatograms of a FSME-standard (A) and from freeze-dried larvae (B) are shown.

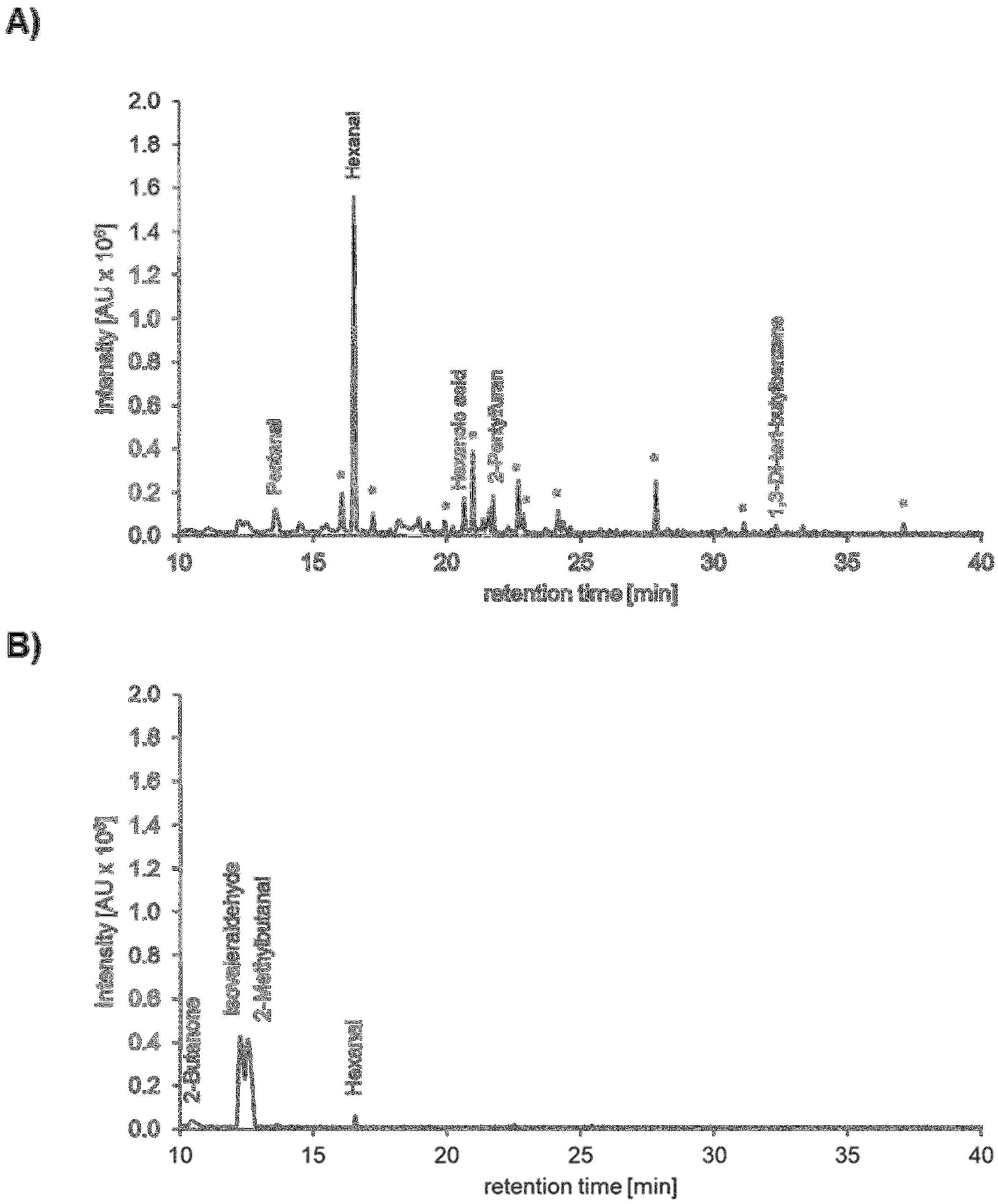

Suppl. Fig. 2. GC-chromatograms from wheat bran (A) and *Tenebrio molitor* larvae (B).

Suppl. Tab. 1. Tabular representation of Headspace GC-analysis of dried *Tenebrio molitor* larvae

Rack oven dried		Freeze dried		Vacuum dried	
**compound**	**Peak area [AU*10^6]**	**compound**	**Peak area [AU*10^6]**	**compound**	**Peak area [AU*10^6]**
**2-Butanone**	0.66 ± 0.01	**Isovaleraldehyde**	18.83 ± 1.84	**2-Butanone**	0.64 ± 0.01
**Isovaleraldehyde**	0.78 ± 0.02	**2-Methylbutanal**	9.56 ± 0.39	**Isovaleraldehyde**	20.24 ± 0.62
**2-Methylbutanal**	1.28 ± 0.06	**2-Methylpropanoic acid**	0.96 ± 0.09	**2-Methylbutanal**	15.97 ± 0.42
**Hexanal**	0.31 ± 0.04	**Hexanal**	0.73 ± 0.06	**Pentanal**	2.90 ± 0.15
**2-Heptanone**	0.21 ± 0.01	**Isovaleric acid**	0.44 ± 0.05	**Hexanal**	8.37 ± 1.53
**2,5-Dimethylpyrazin**	0.27 ± 0.01	**2-Methylbutanoic acid**	0.13 ± 0.01	**2-Heptanone**	0.61 ± 0.01
		**2,5-Dimethylpyrazin**	0.42 ± 0.01	**Heptanal**	0.28 ± 0.02
		**1,3-Di-tert-butylbenzene**	0.01 ± 0.01	**2,5-Dimethylpyrazin**	0.12 ± 0.01
				**Hexanoic acid**	0.50 ± 0.11
				**2-Pentylfuran**	0.29 ± 0.47
				**2-Butyl-2-octenal**	0.33 ± 0.03

Data are shown as means ± SEM of three replicates.
